# The impact of bilingualism in within-language conflict resolution: an ERP study

**DOI:** 10.3389/fpsyg.2023.1173486

**Published:** 2023-05-25

**Authors:** Filip Andras, María Ángeles Ramos, Pedro Macizo

**Affiliations:** ^1^Mind, Brain and Behaviour Research Centre (CIMCYC), Granada, Spain; ^2^Department of Experimental Psychology, University of Granada, Granada, Spain; ^3^Institut Nacional d’Educació Física de Catalunya (INEFC), University of Barcelona, Barcelona, Spain

**Keywords:** bilingualism, cognitive control, conflict resolution, within-language conflict, inhibition

## Abstract

We compared Spanish (L1)–English (L2) bilinguals and Spanish monolinguals in a semantic judgment relationship task in L1 that produced within-language conflict due to the coactivation of the two meanings of a Spanish homophone (e.g., “hola” and “ola” meaning “hello” and “a wave” in English). In this task, participants indicated if pairs of words were related or not (“agua-hola,” “water-hello”). Conflict arose because a word (“agua,” “water”) not related to the orthographic form of a homophone (“hola,” “hello”) was related to the alternative orthographic form (“ola,” “wave”). Compared to a control condition with unrelated word pairs (“peluche-hola,” “teddy-hello”), the behavioral results revealed greater behavioral interference in monolinguals compared to bilinguals. In addition, electrophysiological results revealed N400 differences between monolinguals and bilinguals. These results are discussed around the impact of bilingualism on conflict resolution.

## 1. Introduction

The aim of this paper is to examine whether speaking more than one language has implications for the way people exert cognitive control and manage conflict situations when working in their native language (Spanish, in our case). In the last years, these possible consequences associated with bilingualism have been addressed in many studies with the term “the bilingual advantage” (see Poarch and Krott, 2019, for a review, and Ware et al., 2020 for a meta-analysis with 170 studies on the topic). For the sake of consistency with all these previous studies, in our work we adopt the term “bilingual advantage” (e.g., Morton and Harper, 2007; Costa et al., 2009; Hilchey and Klein, 2011; Paap and Greenberg, 2013; Coderre and van Heuven, 2014; Paap and Liu, 2014; de Bruin et al., 2015; Paap et al., 2015) although we are aware and acknowledge that the more recent terms “bilingual experience” and “language experience” may be preferable to refer to this issue (e.g., Dussias et al., 2019; Torregrossa et al., 2021; Navarro et al., 2022). Moreover, the amount of research on the subject has been extremely abundant in recent years. In our study, we briefly focus on the possible benefits associated with bilingualism and refer the reader to reviews and meta-analyses on the topic.

The possible “advantage” in cognitive control associated to the use of several languages is one of the most controversial issues in current scientific research (for additional reviews see Hilchey and Klein, 2011; Kroll and Bialystok, 2013; Paap and Greenberg, 2013; Lehtonen et al., 2018). Miyake et al. (2000) proposed three main executive functions, namely inhibition of dominant responses, shifting of mental sets, and monitoring and updating of information in working memory. In the current study, we focused on inhibition. In particular, we evaluated the possible “bilingual advantage” in language processing with behavioral and electrophysiological measures of conflict resolution that can be attributed to differences in the inhibitory control of language.

Bilingual speakers are continuously using more than one language, activating representations from both languages which compete against each other when they want to communicate in only one language (Hsieh et al., 2017). Specifically, in the bilingual lexical processing model (inhibitory control model), Green (1998) proposes that all the words known by a bilingual person would contain a language tag specifying the language to which they belong to. Bilinguals performing a task in a target language would activate lexical representations across their languages. Due to this linguistic coactivation, a lexical competition process would take place, which would be solved by suppressing words by virtue of their non-target language tag, thereby allowing words in the target language to be selected. According to this continuous practice in the suppression of competing lexical items, it has been proposed that bilinguals would have an advantage in conflict resolution and inhibitory control compared to monolingual speakers (Hussey et al., 2017).

Furthermore, according to Green (1998), the suppression of lexical information would be carried out by a domain-general inhibitory mechanism. This assumption has led to the proposal that the advantage of bilinguals in cognitive control would extend to other non-linguistic domains such as visual perception (Wimmer and Marx, 2014), the processing of ambiguous figures (Bialystok and Shapero, 2005), conflict resolution and attentional switching (Ooi et al., 2018), working memory (Bialystok et al., 2004), false belief tasks (Rubio-Fernández, 2017), and selective attention (Chung-Fat-Yim et al., 2017) among others. However, empirical evidence shows data both for and against an improved cognitive control in bilinguals compared to monolinguals.

### 1.1. Evidence for and against the “bilingual advantage”

There are several behavioral studies supporting the bilingual advantage in conflict resolution. One of the first works on this topic was reported by Bialystok et al. (2004). The authors compared a group of middle-aged bilinguals, another group of older bilinguals, and a group of monolinguals (matched in age) in various Simon-type cognitive control tasks. In these tasks, participants had to press a key with their right or left hand depending on the color of a square displayed on the computer screen. In the congruent condition, the side in which the square was presented coincided with the response hand (e.g., red square presented on the left and red-left hand). In the incongruent condition, the position of the square did not match the response hand (e.g., red square presented on the right and red-left hand). In general, middle-aged and older bilinguals compared to monolingual speakers showed less conflict effect when they responded to the incongruent condition relative to the congruent condition.

However, the study by Bialystok et al. (2004) presented some limitations such as the limited number of observations used in the Simon task, etc. (see Hilchey and Klein, 2011, for a critical review). Moreover, there are abundant data that do not seem to confirm a bilingual advantage in conflict resolution. For example, using the same Simon task described above, Morton and Harper (2007) found equal conflict resolution in a group of bilinguals and monolinguals (see also Bellegarda and Macizo, 2021). Moreover, the absence of a bilingual advantage in cognitive control has been consistently shown in other studies with the Simon task and other tasks such as the Flanker task, the Card sorting task, etc. (e.g., Paap and Greenberg, 2013; Gathercole et al., 2014, etc.). In addition, recent meta-analysis and theoretical review studies conducted with behavioral data (latency and accuracy measures) seem to find reduced evidence for a bilingual advantage. For example, Lehtonen et al. (2018) synthesized 152 studies comparing monolinguals and bilinguals in six executive domains (inhibition, shifting, working memory, monitoring, attention and verbal fluency). The authors observed no evidence for benefits associated to bilingualism in any of these domains. In addition, Donnelly et al. (2019) reported a multiverse meta-analysis of global reaction time (RT) and interference cost comparing the performance of monolinguals and bilinguals on non-verbal interference control tasks (80 studies). The results revealed a bilingual advantage for global RT and interference cost. However, although significant, this bilingual benefit was very small (see Grundy, 2020 for a Bayesian analysis of 167 independent studies about the effect of bilingualism on executive functions).

As a result of this controversial pattern of data, the debate on the existence of the bilingual advantage in conflict resolution is open. In fact, it has even been proposed that there is a publication bias that would promote the publication of studies in favor of a bilingual advantage (de Bruin et al., 2015; Paap et al., 2015, but see Bialystok et al., 2015). Additionally, Hilchey and Klein (2011) suggested that bilingualism would not be associated to an efficient conflict resolution but to a global advantage in RTs (i.e., including conflicting and non-conflicting trials) because bilinguals would process situations that contain conflict trials differently than monolinguals (e.g., enhanced conflict monitoring skills, Costa et al., 2009). Moreover, it has been proposed that the possible bilingual advantage would depend on the type of bilingual experience and, especially, on the type of conflict situations that bilinguals resolve on a daily basis (Green and Abutalebi, 2013; Fricke et al., 2019; Beatty-Martínez et al., 2020).

Thus, it may be that the better resolution of conflict situations in bilinguals than in monolinguals is not found in tasks that involve conflict resolution in the non-linguistic domain but in situations that entail linguistic conflict. This type of language conflict will be discussed in the next section. However, to anticipate, there are data for and against the impact of bilingualism in both non-linguistic as well as linguistic conflict tasks. For example, previous studies show that bilinguals vs. monolinguals perform better on both non-linguistic tasks (e.g., Simon task, Bialystok et al., 2008) and linguistic conflict tasks (in visual comprehension tasks, e.g., Macizo et al., 2010, and auditory comprehension, Blumenfeld and Marian, 2011). However, other works show no differences between monolinguals and bilinguals in non-linguistic conflict tasks (e.g., Simon task, Duñabeitia et al., 2013) and tasks that requires the resolution of linguistic conflict (i.e., visual and auditory inhibition of irrelevant information; Desjardins and Fernandez, 2018).

### 1.2. The possible “bilingual advantage” in language processing

The fundamental assumption underlying the bilingual advantage in conflict resolution is the idea that the improved cognitive control of bilinguals derives from the continuous linguistic conflict they have to resolve due to the coactivation of their languages (Bialystok et al., 2004). Thus, the bilingual advantage in inhibitory control would be more easily observed when they resolve conflict in tasks that involve language processing.

Duñabeitia et al. (2013) compared a large sample of monolingual and bilingual children during the resolution of a linguistic conflict task in their first language (L1). The authors used a Stroop task in which participants named the color of the ink of words in a congruent condition (the word “red” written in red ink) and an incongruent condition (the word “red” written in green ink). The magnitude of the Stroop-like interference effect (RTs in the incongruent condition relative to the congruent condition) was similar in the bilingual and monolingual group of speakers. Thus, this study revealed that the bilingual advantage is not observed even when conflict resolution refers to linguistic material.

It could be argued that the behavioral measures (e.g., RTs) used in the study conducted by Duñabeitia et al. (2013) were not sensitive enough to capture possible differences in conflict resolution between bilingual and monolingual individuals. For example, Kousaie and Phillips (2012) did not observe behavioral differences between participants when they performed a Stroop task. However, at the electrophysiological level, bilingual people showed smaller N200 amplitude than the monolingual group. This effect was interpreted as evidence that bilinguals required less active conflict monitoring than the monolinguals in order to perform the Stroop task. Thus, the results of this study suggest that in the absence of behavioral differences, an attenuation of ERP components were related to a more efficient processing probably because less resources are needed to achieve the same result (Barulli and Stern, 2013). In our study, we considered both behavioral and electrophysiological measures with the aim of obtaining a complete profile of the possible differences in conflict resolution associated to bilingualism (see Bellegarda and Macizo, 2021, for review and an electrophysiological study with the Stroop task in monolinguals and bilinguals; see Heidlmayr et al., 2020, for a review of the electrophysiological substrates underlying the Stroop task; see Antoniou, 2023, and Cespón and Carreiras, 2020, for reviews of electrophysiological evidence for a possible bilingual advantage in executive functions).

In our opinion, the drawback of using the Stroop task to evaluate the bilingual advantage in conflict resolution is that it does not reflect the type of conflict that bilinguals face when using their two languages. In particular, the conflict in the incongruent condition of the Stroop task comes, at the encoding stage, from two perceptual dimensions (the word and the color of the ink) which activate two different representations that competes for selection (the meaning of two colors) (stimulus-stimulus conflict). Furthermore, the Stroop task involves conflict between the word meaning and the response at the response selection stage (stimulus-response conflict) (De Houwer, 2003). On the contrary, the conflict that arises when bilingual individuals manage two languages usually involves the same dimension of the stimulus such as the processing of interlingual homographs with identical orthography but different meanings across languages. In fact, many studies have observed that when bilinguals understand interlingual homographs, there is conflict due to the concurrent activation of the two meanings of the ambiguous word (Macizo et al., 2010; Martín et al., 2010; Hoshino and Thierry, 2012; Durlik et al., 2016). Furthermore, it is worth noting that while the Stroop task involves speech production (e.g., naming the color of the ink in which words are written), many of the studies showing the effect of bilingualism on language conflict resolution are observed in language comprehension tasks (e.g., Macizo et al., 2010; Martín et al., 2010).

To illustrate, Macizo et al. (2010) used a semantic decision task in which Spanish (L1)–English (L2) bilinguals decided if L2 word pairs were related in meaning. In the critical condition, an interlingual homograph was presented (the word “pie,” meaning “foot” in Spanish and “cake” in English) paired with a word related to the L1 meaning of the homograph (e.g., “pie-toe”). Participants showed worse performance in the critical condition (“pie-toe”) than in a control condition with unrelated word pairs (“pie-log”). Thus, this study indicated that bilinguals coactivated linguistic information across their languages and they had to apply inhibitory control to resolve conflict derived from this coactivation.

From the perspective of the bilingual advantage in conflict resolution, one would expect that in tasks with ambiguous words as that described by Macizo et al. (2010) with interlingual homographs, bilinguals would experience less conflict than monolinguals due to the continued practice of bilinguals with this type of conflicting situation. In our study, we directly evaluated this prediction. However, bilinguals and monolinguals cannot be compared in a between-language task with ambiguous words because monolingual speakers only know one language. Thus, in our experiment, we designed a new paradigm to index linguistic conflict when bilinguals and monolinguals performed a within-language task.

In a behavioral study, Paap and Liu (2014, Experiment 1) directly compared English monolinguals and bilinguals with English as L2 when they processed ambiguous words (homographs) in a within-language task. Following the paradigm used by Gernsbacher et al. (1990, Experiment 4), participants were given English sentences that could end with a homograph or a control word (e.g., “He dug with the spade/shovel”). Afterward, a test word appeared and the participants had to indicate if this word matched the meaning of the sentence just read. In the critical condition, the sentence ended with a homograph was followed by a test word that was not related to the sentence meaning but related to the alternative meaning of the homograph (e.g., “He dug with the spade” ACE). This condition was compared with the one in which the homograph did not appear (e.g., “He dug with the shovel” ACE). The RTs were slower when participants rejected the test word (ACE) in sentences with homographs (spade) compared to sentences with control words (shovel). However, the magnitude of this interference effect was greater in bilinguals than in monolinguals. Thus, a bilingual disadvantage was found.

While the authors accepted the involvement of inhibitory control to suppress the irrelevant homograph meaning in this task (Paap and Liu, 2014, p. 63), the bilingual disadvantage observed in their study was interpreted in terms of the lexical quality hypothesis (Hart and Perfetti, 2008). According to this hypothesis, the ease with which words are processed is determined by the experience people have with them. Thus, bilingual vs. monolingual people would have less experience with English words (words in their L2), which would make more difficult the processing of sentences containing an ambiguous word vs. a control word. However, in our opinion, the Paap and Liu experiment presents some limitations for the study of the possible bilingual advantage in inhibitory control when participants resolve conflict in a within-language task. On the one hand, the experimental task employed by Paap and Liu was conducted in English, and the authors reported differences in fluency between English monolinguals and L2-English bilinguals (p. 56). Thus, despite the presence of inhibitory processes in the task, between-group differences could be explained only by the lexical quality hypothesis, because monolinguals performed the task in their native language and bilinguals in their L2. On the other hand, the authors compared the performance of the participants in trials with/without ambiguous words. However, this comparison would reflect both the interference derived from the coactivation of the two homograph meanings and the functioning of the inhibitory mechanism used to suppress the activation of the homograph irrelevant meaning.

In our study, we attempt to address these two issues. First, we compared Spanish monolinguals and Spanish-English bilinguals performing the linguistic task in their first language (L1). Second, we compared two conditions with ambiguous words (homophones) so that both conditions involved the coactivation of the two homophone meanings. The only difference between conditions was the occurrence of conflict in one of them, thus isolating the inhibitory mechanism used to resolve the conflict in the within-language task.

We acknowledge that the coactivation of words across languages produce between-language conflict because the same meaning (e.g., HOUSE) has two competing lexical forms in each of the bilinguals’ languages (“casa” in Spanish, “house” in English) that compete for selection (e.g., Green, 1998). This between-language conflict may be more frequent than that caused by homonym words (homophones/homographs) within a language (within-language conflict) in which virtually the same lexical forms (phonological/orthographic) (hola/ola in Spanish, “hello” “wave” in English) would coactivate two meanings competing for selection. However, the within-language conflict (the task used in our study, see next subsection) has the main advantage of allowing to evaluate language conflict in monolinguals and bilinguals (all participants resolving language conflict in their native language), thus isolating the possible contribution of the language experience of bilinguals vs. monolinguals in a language conflict task (note that between-language conflict cannot be examined in monolinguals as they only handle their native language). Moreover, homonym words within a language pose a challenge for bilinguals in their everyday life. In fact, it has been observed that when bilinguals are asked to translate homonym words, they are slower and less accurate than in the case of unambiguous words (e.g., Laxén and Lavaur, 2010). Further, homonym words in one language rarely correspond to a single word in another language (Degani and Tokowicz, 2010), and these multiple-translation of ambiguous words produces a disadvantage in terms of latency and accuracy when translating them compared with unambiguous translations (Boada et al., 2013).

### 1.3. The current study

The controversy surrounding the impact of bilingualism on conflict resolution may be in part a result of experimental design and test sensitivity used across studies. The objective of our study was to evaluate the possible benefit of bilingualism in conflict resolution taking into account possible drawbacks of previous studies on the subject. We compared monolinguals and bilinguals in the resolution of a type of conflict that was similar to the one that bilinguals experience on a daily basis due to the coactivation of their languages. Electrophysiological recordings were also considered with the aim of avoiding the possible lack of sensitivity of behavioral measures to index between-group differences in inhibitory control (i.e., Kousaie and Phillips, 2012). However, the predictions in our study were established from a behavioral approach rather than from electrophysiological terms. The reason for this was due to: (a) in conflict tasks, the electrophysiological pattern is not entirely consistent, both in the components that are sensitive to conflict (N2, P3, N400) and in the pattern of results found in each of them. Thus, it is difficult to establish specific predictions. For example, Coderre and van Heuven (2014), using the Stroop task, found no differences between monolinguals and bilinguals on the electrophysiological N400 component. Kousaie and Phillips (2012) observed reduced N2 in bilinguals vs. monolinguals but no between-group differences on the P3 when participants performed the Stroop task. In contrast, the Stroop P3 amplitude was larger in bilinguals in the study by Kousaie and Phillips (2017), (b) the behavioral prediction of the bilingual experience in conflict resolution is parsimonious (lower conflict in bilinguals vs. monolinguals), (c) the conflict task used in our study is not comparable to other conflict tasks employed in previous studies (see next paragraph) so we prefer to make easy-to-understand behavioral predictions.

To evaluate conflict resolution in both Spanish (L1)–English (L2) bilinguals and Spanish monolinguals, we designed a task with within-language ambiguous words in L1. In this task, both bilinguals and monolinguals decided if pairs of L1 words were related or not. The critical stimuli were homophones, words with the same phonology (e.g., the phonological form /’o.la/, International Phonetic Association [IPA], 1999), but two different meanings associated to their two possible orthographic forms (e.g., “ola,” “a wave” in English and “hola,” “hello” in English). Please note that homophone words differ in meaning, although they may also differ in spelling. Spanish, however, is a transparent language with a high degree of grapheme to phoneme correspondence. Thus, the homophone /’o.la/ has a single phonological form and only one grapheme that differentiates its two orthographic forms (i.e., “h” in the example). Therefore, the main characteristic of these words is the presence of a very similar superficial (phonological/orthographic) form but two completely different meanings.

Two conditions were implemented in our study. In the related condition, one orthographic form of the homophone (e.g., “hola,” “hello” in English) was preceded by a word that was not related to this orthographic form (“agua,” “water” in English) but produced conflict because it was related to the alternative orthographic form of the homophone (e.g., “ola,” “wave” in English). In the unrelated condition, the homophone was paired with a word, presented before the homophone, that was unrelated to either of the two orthographic forms of the homophone (e.g., “peluche,” “teddy” in English). In this task, the conflict associated to within-language coactivation would be reflected in a worse performance in the related condition relative to the unrelated condition. Critically, if there is a bilingual advantage in inhibitory control when bilinguals experience conflict in the linguistic domain, the magnitude of the conflict effect (related vs. unrelated trials) would be smaller in bilinguals than in monolinguals.

## 2. Materials and methods

### 2.1. Participants

Sixty Spanish university students took part in the study voluntarily. They gave written informed consent before performing the experiment. The study was approved by the ethical committee at the University where the experiment was conducted (number issued by the ethical committee: 957/CEIH/2019). The participants reported no history of language disabilities and they had normal or corrected-to-normal visual acuity. All students were native speakers of Spanish. Twenty-eight participants were Spanish monolinguals (13 men and 15 women) and thirty-two participants were Spanish (L1)–English (L2) bilinguals (15 men and 17 women).

It is practically impossible to find a group of “pure” monolinguals in Spain without any contact with the English language. However, we used strict inclusion criteria to conform the two groups of participants in our study. The inclusion criterion for the bilingual group was that they had a B2 or higher level of English as a foreign language according to the Common European framework of reference for languages: Learning, teaching, assessment (CEFR, Council of Europe, 2001). On the other hand, the inclusion criterion to be part of the monolingual group was that the participants did not have any knowledge of English as L2 or a level lower than B1.

The required sample size was determined using the G*Power program 3.1.9.2 (Faul et al., 2007). To achieve a 95% statistical power at α = 0.05 and a small effect size (0.20) computed based on a η_*p*_^2^ = 0.15, in a 2 × 2 mixed design with relatedness (related, unrelated) as the within-participants factor and group (monolinguals, bilinguals) as the between-participants factor, the total sample size required in our study was *N* = 58 (29 monolinguals, 29 bilinguals). Thus, the sample used in this study was sufficient to capture the effects evaluated in the experiment.

At the end of the experimental session, the participants were asked to complete the “language experience and proficiency questionnaire” (LEAP-Q) (Marian et al., 2007) to evaluate their language proficiency on reading, writing, listening and speaking in Spanish (L1) and English (L2). We decided to use the LEAP-Q questionnaire in our study because their values have been shown to correlate with standardized measures of language skill in L1 and L2. Thus, Marian et al. (2007) revealed that the self-rated measures of language fluency obtained by the LEAP-Q questionnaire correlated with eight objective measures of language processing (Pearson *r* values ranging from 0.29 to 0.74).

**FIGURE 1:**
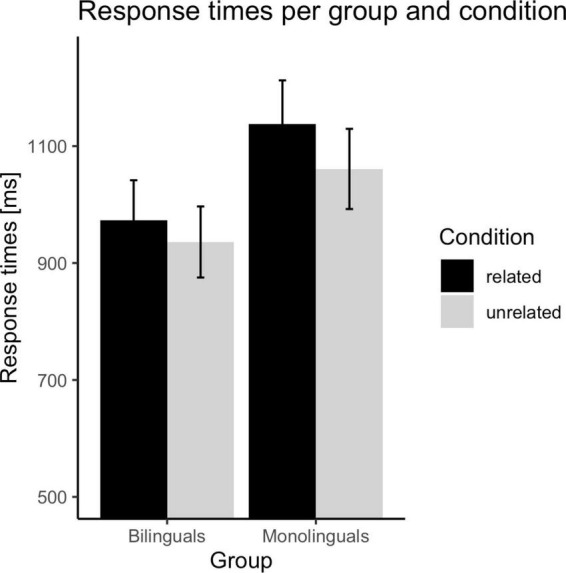
Mean response times per group and condition. The error bars display a 95% confidence interval.

**FIGURE 2:**
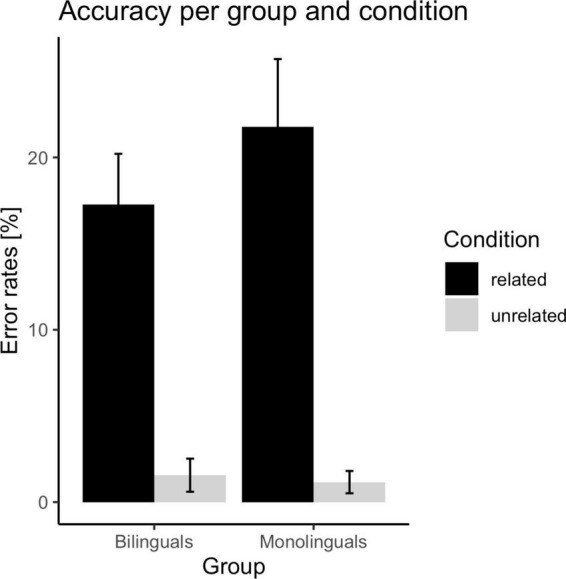
Mean error rates per group and condition. The error bars display a 95% confidence interval.

Both bilinguals and monolinguals were matched on Spanish (L1) linguistic skills, and bilinguals were fluent in English. The average fluency in L2 of the bilingual group was higher (*M* = 7.53, *SD* = 1.14) than that obtained by the monolingual group (*M* = 4.35, *SD* = 1.77), *t*(58) = 8.40, *p* = 0.002. Furthermore, the percentage of exposure to L2 was nearly double in the bilingual group (29.74%) than in the monolingual group (15.85%). The participant’s characteristics are reported in Table 1. The mean participants response times and error rates can be found in Figures 1, 2.

**TABLE 1:** Characteristics, language proficiency and language use of participants in the study.

	Bilinguals	Monolinguals	*t*(58) values
Age (years)	23.34 (3.81)	24.07 (3.71)	
Age starting L2 learning (years)	5.84 (2.17)	7.46 (2.81)	
Living in L2 speaking countries (months)	6.58 (15.77)	0.43 (1.26)	
Age becoming fluent in L2 (years)	15.77 (4.74)		
**Language proficiency questionnaire**			
L1 speech fluency	9.41 (0.61)	8.71 (1.01)	3.14, *p* = 0.003*
L1 speech comprehension	9.56 (0.72)	9.29 (0.85)	1.37, *p* = 0.18
L1 reading proficiency	9.44 (0.76)	9.29 (0.71)	0.80, *p* = 0.43
L1 proficiency	9.47 (0.61)	9.10 (0.75)	2.12, *p* = 0.04*
L2 speech fluency	7.19 (1.31)	3.43 (1.89)	8.83, *p* < 0.001**
L2 speech comprehension	7.50 (1.59)	4.46 (2.12)	6.21, *p* < 0.001**
L2 reading proficiency	7.91 (1.17)	5.14 (2.07)	6.25, *p* < 0.001**
L2 proficiency	7.53 (1.14)	4.35 (1.77)	8.17, *p* < 0.001**
**Language use**			
Current exposure to L1	71.5	82.61	−2.35, *p* = 0.02*
Preference for reading in L1	59.34	84.71	−4.31, *p* < 0.001**
Preference for speaking with another person in L1	59.38	80.18	−3.16, *p* = 0.003*
Time interacting with friends in L1	88.13	97.14	−2.16, *p* = 0.04*
Time interacting with family in L1	91.56	96.79	−1.19., *p* = 0.24
Time reading in L1	69.38	82.14	−2.00 *p* = 0.05*
Time watching TV in L1	62.19	80.71	−2.43, *p* = 0.02*
Time listening to the radio in L1	55.31	64.29	−1.43, *p* = 0.16
Current exposure to L2	29.74	15.85	3.26, *p* = 0.002*
Preference for reading in L2	40.71	12.88	4.98, *p* < 0.001**
Preference for speaking with another person in L2	38.00	17.62	3.10, *p* = 0.003*
Time interacting with friends in L2	34.69	22.50	1.70, *p* = 0.1
Time interacting with family in L2	5.94	3.93	0.56, *p* = 0.58
Time reading in L2	62.19	50.71	1.88, *p* = 0.07
Time watching TV in L2	64.69	34.29	4.36, *p* < 0.001**
Time listening to the radio in L2	66.88	60.71	0.98, *p* = 0.33

Mean values and standard errors (in parenthesis) of the participants characteristics and the language proficiency questionnaire. Scales in the language proficiency questionnaire range from 0 (lowest score) to 10 (highest score). Proficiency refers to the mean proficiency of the other three measures (speech fluency, speech comprehension and reading proficiency). The values of the language use scales are given in percentages. *p < 0.05, **p < 0.001.

As can be seen in Table 1, between-group differences were observed in speech fluency in L1, with lower L1 speech fluency in monolinguals than in bilinguals. These differences were not expected. Although we do not have a satisfactory explanation for these differences, we do not anticipate that they determined the pattern of results because the task was conducted in visual format in the native language of the participants and there were no differences between monolinguals and bilinguals in L1 reading proficiency (*p* = 0.43). Furthermore, in the hypothetical case that L1 speech fluency modulated conflict resolution, it would be expected that greater verbal fluency would produce more within-language coactivation in the native language of bilinguals which would be associated with more conflict compared to monolinguals (contrary to the predictions of our study).

### 2.2. Design and materials

The stimuli, experimental task, and Supplementary material used in the current study are freely available at https://osf.io/amwn2/. In the current experiment, we designed a homophone task to index within-language conflict that could be performed by bilinguals and monolinguals. The task was conducted in the participants’ L1 (Spanish). In this task, participants received pairs of words and they had to indicate whether they were semantically related or not. The task was composed of 40 Spanish homophones (see Supplementary material 1 for the complete set of material). The 40 homophones were words with a single Spanish phonology but two possible meanings depending on their orthographic form. For example, the phonological word /k a ʝ a ð o/ (International Phonetic Association [IPA], 1999), has the meaning of “silent” (Real Academia Española, 2017), when it is associated to one orthographic form “callado” (homophone 1), but it has the meaning of “crook” when it is associated to the alternative orthographic form “cayado” (homophone 2).

The 40 homophones were presented in a related condition and in an unrelated condition. In the related condition, an orthographic form of the homophone (e.g., homophone “cayado,” “crook” in English) was paired with a word related to the meaning of the alternative orthographic form of the homophone (“ruidoso,” “noisy” in English, associated to the orthographic form of the homophone “callado,” “silent” in English) (see Table 2 for examples). In the unrelated condition, each homophone was paired with a word that was not related to either of the two orthographic forms of the homophone (e.g., “cayado”–“película”; “crook”–“film” in English).

**TABLE 2:** Examples of the pairs of words in each condition of the homophone task.

Related condition	Unrelated condition
agua–hola (water–hello) *ola (a wave)*	Peluche–hola (teddy–hello)
ruidoso–cayado (noisy–crook) *callado (silent)*	Película–cayado (film–crook)

The homophone task was conducted in the participants’ L1 (Spanish) (approximate English translation is given in parenthesis). The alternative orthographic form of the homophone is given in Italics. The word pairs were presented in sequential order on each trial. First, participants received the related word (e.g., “agua,” “water” in English) or the unrelated word (e.g., “peluche,” “teddy” in English) depending on the experimental condition. Next, the homograph word (“hola,” “hello” in English) was appeared and participants had to indicate whether the homograph word was related or not to the previously presented word (see Section “2.3. Procedure,” for additional details).

The associative strength between the associated word (e.g., “agua,” “water” in English) and the orthographic form of the homophone related to the associated word (“ola,” “wave” in English) (forward strength from cue, e.g., “agua” to target, e.g., “ola”) was obtained from the free association norms in Spanish (the language used in our study). This database of Spanish words was developed and is freely available at the University of Salamanca, Spain (NALC) (Fernández et al., 2013). These norms include 6,739 words, obtained from a set of 2,305 young students (*M* age = 19.6) in a period of 16 years. To our knowledge, this is the most complete associative database in Spanish at present. In the related condition, the forward associative strength was *M* = 0.12, *SD* = 0.14 (values expressed in proportions). In the unrelated condition the associative strength was *M* = 0.00.

In the unrelated condition, the 40 homophones were paired with 40 non-homophone words that were not related to either of the two spellings of the homophones (the associative strength value of the unrelated pairs was equal to 0). The word-form similarity between the homophone word and the non-homophone word calculated with Levenshtein’s distance (1966) and it was equated in the related condition (*M* = 5.05, *SD* = 1.45) and the unrelated condition (*M* = 5.22, *SD* = 1.02), *t*(39) = 0.64, *p* = 0.53. The 40 non-homophone words of the related and unrelated condition were matched in length (mean number of letters) (related condition, *M* = 5.80, *SD* = 1.56, unrelated condition, *M* = 5.88, *SD* = 1.32, *t*(39) = 0.24, *p* = 0.81), and lexical frequency (per one million count, Cuetos et al., 2011) (related condition, *M* = 77.68, *SD* = 120.74, unrelated condition, *M* = 160.05, *SD* = 391.47), *t*(39) = 1.23, *p* = 0.22. Additionally, the non-homophone words in the related and unrelated condition were equated in different lexical and sublexical parameters in Spanish (B-Pal software, Davis and Perea, 2005) see Table 3.

**TABLE 3:** Characteristics of stimuli used in the study.

	Homophone words vs. Non-homophone words		
	**Related condition**	**Unrelated condition**	***t*(39) values**
Forward associative strength	0.12 (0.14)	0.00	
Levenshtein’s distance	5.05 (1.45)	5.22 (1.02)	0.64, *p* = 0.53
**Non-homophone words**			
Length (number of letters)	5.80 (1.56)	5.88 (1.32)	0.24, *p* = 0.81
Word frequency	77.68 (120.74)	160.05 (391.47)	1.23, *p* = 0.22
Concreteness	3.22 (2.38)	4.00 (2.36)	1.40, *p* = 0.17
Familiarity	4.16 (2.97)	4.66 (2.62)	0.72, *p* = 0.48
Imaginability	3.49 (2.69)	4.42 (2.55)	1.45, *p* = 0.16
Bigram frequency (log)	2.58 (0.45)	2.66 (0.32)	0.92, *p* = 0.36
Orthographic neighbors (ON)	4.48 (5.47)	4.25 (5.65)	0.20, *p* = 0.85
ON frequency	26.69 (56.63)	19.33 (34.04)	0.69, *p* = 0.49
Phonological neighbors (PN)	6.49 (7.04)	5.59 (7.82)	0.57, *p* = 0.57
Mean frequency of PN	39.09 (124.14)	19.99 (41.01)	0.86, *p* = 0.39

Characteristics of experimental stimuli used in the study (mean values and standard deviation in parenthesis). Forward Associative strength between the related word (cue) and the orthographic form of the homophone (target) related with the preceding cue word (Fernández et al., 2013) (values expressed in proportions). Levenshtein’s distance (Levenshtein, 1966) is the minimum number of single-character edits (insertions, deletions or substitutions) required to change one word into the other. Length is given in number of letters. Word frequency per one-million count (Cuetos et al., 2011). Concreteness, familiarity and imageability subjective ratings obtained from the LEXESP (a database based on approximately 5 million Spanish words, Sebastián-Gallés et al., 2000). Spanish database on a scale from 1 to 7, where higher scores indicate greater value. Bigram frequency: Mean logarithmic bigram frequency based on the LEXESP word frequency corpus. Orthographic neighbors (ON): Orthographic neighborhood size determined by counting the number of words that can be formed by substituting a single letter at any of the letter positions within the word. ON frequency: The average frequency of the word’s orthographic neighbors. Phonological neighbors (PN): Mean number of phonological neighborhood. PN frequency: The average frequency of the word’s phonological neighbors.

### 2.3. Procedure

The study was conducted in a single session. Participants were tested individually in the EEG recording room. E-prime experimental software was used for stimulus presentation and data collection (Schneider et al., 2002). In the experimental task, the participants received the set of 40 experimental trials with homophones twice, once in the related condition and once in the unrelated condition (80 trials in total, 40 related trials, 40 unrelated trials). All experimental trials were associated to “no” responses (the word pairs were unrelated). With the aim of including trials associated to “yes” responses, we added filler trials. These filler trials contained 80 pairs of words, 40 trials with related word pairs (e.g., “agua–río,” “water–river,” in English, “yes responses”) and 40 trials with unrelated word pairs (e.g., “botella–coche,” “bottle–car” in English, “no responses”). Thus, each participant received 160 pairs of words divided into four experimental blocks of 40 trials each with the aim of allowing the participants to rest between each experimental block. Each block of trials consisted of 20 experimental trials (10 related, 10 unrelated) and 20 filler trials (10 related, 10 unrelated). Within a block, a homophone word never appeared twice and trials were randomized within each block.

The percentage of related and unrelated trials determines participants’ performance on, for example, semantic priming tasks (see Den Heyer, 1985, for an early review). However, in our study, the two critical conditions we compared (related and unrelated trials both associated with “no” responses) were matched in number of trials (40 trials in each condition). The inclusion of 80 filler trials (non-homophone trials) (40 “yes” response trials and 40 “no” response trials) were added in order to implement the semantic decision task (deciding whether the word pair was related or not required “yes” and “no” responses). Thus, there was an unequal number of “yes/no” responses in our study. It is true that we could have used the same number of “yes/no” responses by adding more filler trials associated to “yes” responses, but this would increase the duration of the experiment and the fatigue of the participants. In any case, as we indicated, the filler trials are not relevant and were not analyzed as they were not needed to investigate the topic at hand (the effect of language experience in within-language conflict). Importantly the critical trials with homophones (related and unrelated condition) were equated in number and type of response (“no” responses).

All stimuli were presented on a desktop computer screen, in Arial font, 30 point size, white font and black background. On each trial, a fixation point was presented for 1,000 ms in the middle of the screen. After that, the first word of the pair, the related word (e.g., “agua,” “water” in English) or the unrelated word (e.g, “peluche,” “teddy” in English) was presented depending on the experimental condition for 500 ms followed by a black screen for 500 ms. Next, the homophone (e.g., “hola,” “hello” in English) was presented for 500 ms followed by a 2,500 ms time interval. A jittered inter-stimulus interval (ISI) may prevent automatic response from participants. However, we decided to use a fixed ISI similar to other studies about the processing of ambiguous word pairs in bilinguals (Macizo et al., 2010; Martín et al., 2010). Participants had to indicate whether or not the two words were related in meaning by pressing the M and Z keys on the keyboard. The assignment of the M/Z keys to yes/no responses was counterbalanced across participants. It is important to note that the sequence of words within a single trial could have started with the presentation of the homograph word followed by the related or unrelated word (depending on the experimental condition). However, this mode of presentation has the limitation that the words to which the participant responds would not be the same in both experimental conditions. Thus, we decided to first present the related/unrelated words followed by the homograph word to which the participants had to respond in the two experimental conditions.

At the end of the experimental session, participants filled in the LEAP-Q questionnaire (Marian et al., 2007) and were informed about the purpose of the research.

### 2.4. EEG recording and analysis

The continuous Electroencephalogram (EEG) was recorded at a sampling rate of 500 Hz using 64 Ag-Ag-Cl electrodes mounted on a nylon Quik-cap (Compumedics USA, Charlotte, NC, USA) arranged as specified by the extended 10–20 International System (Jasper, 1958). The EEG was initially recorded against an electrode placed in the midline of the cap (between Cz and CPz) and later off-line re-referenced to a common average reference (i.e., average across channels and subtraction of that average potential from each electrode, Nunez et al., 2019). The electro-oculogram (EOG) was bipolarly recorded. In order to record the horizontal EOG, two electrodes were placed on the outer canthus in both eyes. To take measure of the vertical EOG, two electrodes were located in the supra and infra-orbital zone of the left eye. EEG and EOG signals were amplified by using the Neuroscan Synamps2 amplifiers (El Paso, TX, USA) and filtered using a band pass of 0.01–100 Hz. The electrodes impedance was kept below 5 kΩ. Digital tags were assigned to the stimulus of interest.

SCAN 4.3.1 (Compumedics, USA, Charlotte, NC, USA) was used to acquire the EEG. For the offline processing, EEGlab version 2019.0 (Delorme and Makeig, 2004), ERPlab 7.0 (López-Calderón and Luck, 2014) and Matlab R2019a (MATLAB and Statistics Toolbox Release 2019a, The MathWorks, Inc., Natick, MA, USA) were used. This offline processing included applying a low pass 30Hz filter, correcting for eye blinks and horizontal/vertical eye movements as well as other artifacts during the independent component analysis (ICA). Epochs were baseline corrected using the mean activity during the −100 to 0 ms pre-stimuli period.

Statistical analyses were conducted on the average amplitude in four consecutive time-windows of 100 ms each, from 200 ms to 500 ms (200–300 ms, 300–400 ms, and 400–500 ms) which were time-locked to the onset of the second word (the homophone words). These time windows were chosen on the basis of previous studies on the subject (Alvarez et al., 2003; Kerkhofs et al., 2006). The lateral media axis (left, midline and right electrodes) was taken into account on five anterior-posterior regions: Frontal region (F3, Fz, F4), fronto-central region (FC3, FCz, FC4), central region (C3, Cz, C4), centro-parietal region (CP3, CPz, CP4) and parietal (P3, Pz, P4). These electrodes considered were selected according to Luck (2014) (see Tejero and Macizo, 2020, for a similar approximation). According to Luck, even when multiple electrode sites are recorded, it is usually best not to include measurements from electrode sites spanning the entire scalp because sites where an ERP component is not present might add noise. Thus, the electrodes selected for our analyses present a good representation of the topographic distribution of the scalp regions (Luck, 2014).

Analyses were conducted with Group (bilingual group, monolingual group), Relatedness (related condition, unrelated condition), anterior-posterior axis (frontal, fronto-central, central, centro-parietal and parietal) and lateral-medial axis (left, midline and right). For the repeated-measure ANOVAs, the Greenhouse-Geisser correction (Greenhouse and Geisser, 1959) for non-sphericity of variance was used for all *F*-ratios with more than one degree of freedom in the denominator; reported here are the original *df*, the corrected probability level, and the ε correction factor. In addition, Bonferroni correction for multiple comparisons were applied in the analyses reported in text.

## 3. Results

The data and analyses conducted in this study are freely available at https://osf.io/amwn2/.

### 3.1. Behavioral results

Trials on which participants committed an error were eliminated from the latency analysis and submitted to the accuracy analysis in the homophone task performed by bilinguals (10.45%) and monolinguals (12.08%). Furthermore, we excluded RTs below and above 2.5 *SD* for each individual participant’s mean (4.43% in the bilingual group and 4.04% in the monolingual group).

Data analysis was conducted using analysis of variance (ANOVA), given the large number of previous studies using this approach both in favor (e.g., Bialystok et al., 2004) and against (e.g., Duñabeitia et al., 2013) of the impact of bilingualism on cognitive control. However, our data were also analyzed using the mixed-model approach. The pattern of results obtained using these two analyses (ANOVA and mixed-models) was nearly the same. Mixed models analyses and outcomes are available at https://osf.io/amwn2/.

The ANOVAs were conducted with participants (*F*_1_, *t*_1_) and items (*F*_2_, *t*_2_) as random factors. The relatedness (related, unrelated) was considered the within-participants variable and the group (monolinguals, bilinguals) was manipulated between-participants in a 2 × 2 mixed factorial design. The behavioral results obtained in the study are summarized in Table 4.

**TABLE 4:** Behavioral results obtained in the study.

	Related condition		Unrelated condition		Conflict	
	**RT**	**E%**	**RT**	**E%**	**RT**	**E%**
Bilinguals	973 (35)	17.27% (1.50)	936 (31)	1.56% (0.49)	37	15.70%
Monolinguals	1138 (38)	21.79% (2.00)	1061 (35)	1.16% (0.33)	77	20.63%
Group diff.	165	4.52%	125	0.40%		

Mean reaction times (RT, in milliseconds), Error percentages (E%, in percentages) and standard errors (in parenthesis) obtained in the homophone task as a function of relatedness (related condition, unrelated condition) in bilinguals and monolinguals. Conflict: Conflict effect, difference between the related condition minus the unrelated condition. Group Diff.: Average difference between monolinguals minus bilinguals in the related and unrelated conditions.

The ANOVA conducted with RT data revealed a main effect of group, *F*_1_(1,58) = 8.74, *p* = 0.004, η_*p*_^2^ = 0.13, *F*_2_(1,39) = 261.13, *p* < 0.001, η_*p*_^2^ = 0.87. The response time was faster in the group of bilinguals (*M* = 955 ms, *SE* = 33.52) than in the group of monolinguals (*M* = 1100 ms, *SE* = 35.83). The main effect of relatedness was significant, *F*_1_(1,58) = 54.60, *p* < 0.001, η_*p*_^2^ = 0.49, *F*_2_(1,39) = 30.63, *p* < 0.001, η_*p*_^2^ = 0.44. The RTs were slower in the related condition (*M* = 1056 ms, *SE* = 26.18) than in the unrelated condition (*M* = 999 ms, *SE* = 23.42). Finally, the Group x Relatedness interaction was significant in the participants analysis, *F*_1_(1,58) = 6.63, *p* = 0.01, η_*p*_^2^ = 0.10, but not in the items analysis, *F*_2_(1,39) = 2.28, *p* = 0.14, η_*p*_^2^ = 0.06. Planned comparisons revealed that the relatedness effect was significant in the group of bilinguals, *t*_1_(31) = 3.61, *p* = 0.001, *t*_2_(39) = 3.23, *p* = 0.003, and the group of monolinguals, *t*_1_(27) = 6.64, *p* < 0.001, *t*_2_(39) = 4.52, *p* < 0.001. However, the magnitude of the relatedness effect was smaller in the group of bilinguals (37 ms) than in the group of monolinguals (77 ms). In addition, monolinguals were slower than bilinguals in both the related condition, *t*_1_(58) = 3.15, *p* = 0.003, *t*_2_(40) = 9.32, *p* < 0.001, and the unrelated condition, *t*_1_(58) = 2.67, *p* = 0.01, *t*_2_(40) = 12.91, *p* < 0.001. However, the group effect (monolingual vs. bilingual) was greater in the related condition (165 ms difference) than in the unrelated condition (125 ms difference).

The ANOVA conducted with error data revealed that the main effect of group was not significant in the participants analysis, *F*_1_(1,58) = 2.53, *p* = 0.12, η_*p*_^2^ = 0.04, but it was in the items analysis, *F*_2_(1,39) = 7.55, *p* = 0.01, η_*p*_^2^ = 0.16. The percentage of errors committed by bilinguals and monolinguals was *M* = 9.41% (*SE* = 0.88) and *M* = 11.47% (*SE* = 0.95), respectively. The relatedness effect was significant, *F*_1_(1,58) = 215.24, *p* < 0.001, η_*p*_^2^ = 0.79, *F*_2_(1,39) = 43.54, *p* < 0.001, η_*p*_^2^ = 0.53. The participants committed more errors in the related condition (*M* = 19.53%, *SE* = 1.23) than in the unrelated condition (*M* = 1.36%, *SE* = 0.30). Importantly, the Group × Relatedness interaction was significant, *F*_1_(1,58) = 3.95, *p* = 0.05, η_*p*_^2^ = 0.06, *F*_2_(1,39) = 7.18, *p* = 0.01, η_*p*_^2^ = 0.16. Planned comparisons revealed that the relatedness effect was significant in the group of bilinguals, *t*_1_(31) = 9.99, *p* < 0.001, *t*_2_(39) = 5.37, *p* < 0.001, and the group of monolinguals, *t*_1_(27) = 10.61, *p* < 0.001, *t*_2_(39) = 7.16, *p* < 0.001. However, the magnitude of the relatedness effect was smaller in the group of bilinguals (16%) than in the group of monolinguals (21%). On the other hand, in the related condition, the group effect was close to significance in the participant analysis, *t*_1_(58) = 1.84, *p* = 0.07, and it was significant in the item analysis, *t*_2_(40) = 2.87, *p* = 0.007. However, the group effect was not significant in the unrelated condition, *t*_1_(58) = 0.67, *p* = 0.51, *t*_2_(40) = 0.69, *p* = 0.49. Thus, in the related condition, monolinguals committed more errors than bilinguals (4.52% difference), while the accuracy of participants was similar in the unrelated condition (0.40% difference).

### 3.2. Electrophysiological results

**FIGURE 3:**
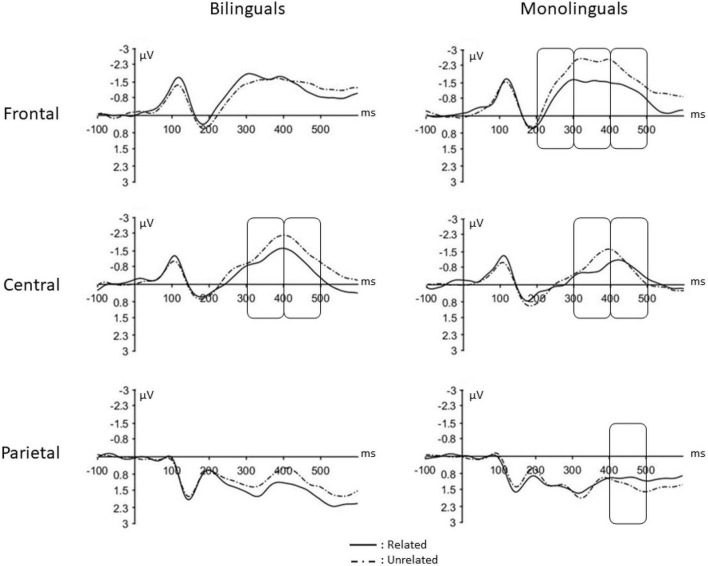
ERPs obtained in the homophone task. Event related potentials (ERPs) obtained in the related and unrelated condition when bilinguals and monolinguals performed the within-language conflict task. Vertical boxes indicate time windows in which the relatedness effect was significant in frontal, central and parietal scalp distributions.

The Group (monolinguals, bilinguals) was entered in the analyses as a between-participants factor along with Relatedness (related, unrelated) × Anterior-posterior axis (frontal, fronto-central, central, centro-parietal, parietal) × Lateral axis (left, midline, right) as within-participants variables. Thus, a 2 × 2 × 5 × 3 mixed factorial design was considered. Figure 3 shows a summary of the relationship effect on bilinguals and monolinguals. The summary of all the statistical analyses including the relatedness factor and its interaction with other variables is reported in Supplementary materials 2, 3 (bilingual group and monolingual group, respectively). The complete pattern of results obtained in the homophone task is visually presented in Supplementary materials 4, 5 (bilingual group and monolingual group, respectively).

#### 3.2.1. 200–300 ms time-window

The Group × Relatedness × Anterior-posterior axis interaction was significant, *F*(4,232) = 2.47, *p* = 0.05, η_*p*_^2^ = 0.04. In frontal regions, the Group × Relatedness effect was significant, *F*(1,178) = 6.13, *p* = 0.01, η_*p*_^2^ = 0.03. The relatedness effect was not significant in bilinguals, *t*(95) = −0.97, *p* = 0.33, but it was in monolinguals, *t*(83) = 2.31, *p* = 0.02, with more positive amplitude in the related condition (*M* = −0.54 μV, *SE* = 0.35) than in the unrelated condition (*M* = −0.94 μV, *SE* = 0.37). However, no differences were observed between bilingual and monolingual in either the related condition *t*(56) = 0.51, *p* = 0.61, or the unrelated condition, *t*(56) = 0.58, *p* = 0.56. In fronto-central regions, central, centro-parietal and parietal regions, the relatedness effect and the Group × Relatedness interactions were not significant (all *p*s > 0.05). Thus, in the 200–300 time window, the relatedness effect was not found in bilinguals, whereas in monolinguals it was significant in frontal regions.

#### 3.2.2. 300–400 ms time-window

The Group × Relatedness × Anterior-posterior axis interaction was significant, *F*(4,232) = 4.14, *p* = 0.003, η_*p*_^2^ = 0.07. In frontal regions, the Group × Relatedness effect was significant, *F*(1,178) = 12.02, *p* = 0.001, η_*p*_^2^ = 0.06. The relatedness effect was not significant in bilinguals, *t*(95) = 0.28, *p* = 0.78, but it was in monolinguals, *t*(83) = 3.94, *p* < 0.001, with more positive brain waves in the related condition (*M* = −1.01 μV, *SE* = 0.27) than in the unrelated condition (*M* = −1.85 μV, *SE* = 0.37). However, there were no between-groups differences in the related condition, *t*(56) = 0.79, *p* = 0.43, or the unrelated condition, *t*(56) = 0.69, *p* = 0.50. In fronto-central regions, the Group x Relatedness effect was significant, *F*(1,178) = 8.40, *p* = 0.004, η_*p*_^2^ = 0.05. The relatedness effect was not significant in bilinguals, *t*(95) = 1.30, *p* = 0.20, but it was significant in monolinguals, *t*(83) = 4.02, *p* < 0.001, with more positive amplitude in the related condition (*M* = −0.88 μV, *SE* = 0.25) than in the unrelated condition (*M* = −1.57 μV, *SE* = 0.24). The difference between bilinguals and monolinguals was significant in the related condition, *t*(56) = 2.00, *p* = 0.05, but it was not significant in the unrelated condition, *t*(56) = 0.70, *p* = 0.48. In central regions, the relatedness effect was significant, *F*(1,178) = 13.46, *p* < 0.001, η_*p*_^2^ = 0.07, but not the Group x Relatedness interaction, *p* > 0.05. In centro-parietal and parietal regions the relatedness effect and the Group x Relatedness interaction were not significant (all *p*s > 0.05). Thus, in the 300–400 time window, the monolingual group showed the relatedness effect in all topographic regions (frontal, fronto-central, central, centro-parietal and parietal regions), while the bilingual individuals showed the effect only in central, centro-parietal and parietal regions.

#### 3.2.3. 400–500 ms time-window

The Group × Relatedness × Anterior-posterior axis × Lateral axis interaction was significant, *F*(8,464) = 2.28, *p* = 0.02, η_*p*_^2^ = 0.04. In frontal regions, the Group x Relatedness effect was significant, *F*(1,178) = 3.60, *p* = 0.06, η_*p*_^2^ = 0.02. The relatedness effect was not significant in bilinguals, *t*(95) = 0.92, *p* = 0.36, but it was in monolinguals, *t*(83) = 3.60, *p* = 0.001, with more positive brain waves in the related condition (*M* = −0.84 μV, *SE* = 0.21) than in the unrelated condition (*M* = −1.40 μV, *SE* = 0.22). However, there were no between-groups differences in the related condition, *t*(56) = 0.56, *p* = 0.58, or the unrelated condition, *t*(56) = 0.14, *p* = 0.89. In fronto-central regions, the relatedness effect was significant, *F*(1,178) = 12.94, *p* < 0.001, η_*p*_^2^ = 0.07, but the Group × Relatedness effect was not significant, *F*(1,178) = 0.51, *p* = 0.48, η_*p*_^2^ = 0.003. In central regions, the relatedness effect was significant, *F*(1,178) = 4.44, *p* = 0.04, η_*p*_^2^ = 0.02, but not the Group × Relatedness interaction, *F*(1,178) = 1.25, *p* = 0.27, η_*p*_^2^ = 0.01. In centro-parietal regions, the Group × Relatedness effect was significant, *F*(1,178) = 5.15, *p* = 0.02, η_*p*_^2^ = 0.03. In the bilingual group, there was a trend toward a relatedness effect, *t*(95) = 1.94, *p* = 0.06, there was more positive brain waves in the related condition (*M* = −0.14 μV, *SE* = 0.22) than in the unrelated condition (*M* = −0.36 μV, *SE* = 0.25). In the monolingual group, the relatedness effect was not significant, *t*(83) = −1.28, *p* = 0.21. However, there were no differences between monolinguals and bilinguals in the related condition, *t*(56) = 0.33, *p* = 0.74, or the unrelated condition, *t*(56) = 0.82, *p* = 0.42. In parietal regions the Group x Relatedness effect was significant, *F*(1,178) = 4.95, *p* = 0.03, η_*p*_^2^ = 0.03. In the bilingual group, the relatedness effect was not significant, *t*(95) = 1.01, *p* = 0.32. In the monolingual group, the relatedness effect was significant, *t*(83) = −2.12, *p* = 0.04, in this case, the brain-waves were more negative in the related condition (*M* = 0.97 μV, *SE* = 0.28) than in the unrelated condition (*M* = 1.24 μV, *SE* = 0.29). However, there were no between-groups differences in the related condition, *t*(56) = 0.94, *p* = 0.35, or the unrelated condition, *t*(56) = 0.04, *p* = 0.97.

Thus, in the 400–500 time window, bilinguals showed the relatedness effect in fronto-central regions only while the effect was broadly distributed in monolinguals across frontal, fronto-central and central regions with more positive amplitudes in the related condition than the unrelated condition. Furthermore, in monolinguals, the relatedness effect was also found in parietal regions; however, in this region, the usual N400 effect was found with more negative amplitudes in the related condition than in the unrelated condition.

In addition, we performed correlation analyses to determine whether the magnitude of the conflict effect found with behavioral measures was associated to modulations in the N400 amplitude obtained with electrophysiological measures. The results of the analysis revealed that the conflict effect (related minus unrelated trials) on latency and response accuracy did not correlate with variations in N400 amplitude (related minus unrelated trials) in any brain region at the 400–500 ms time-window (all *p*s > 0.05).

## 4. Discussion

In recent decades it has been suggested that bilinguals exhibit more efficient conflict resolution than monolinguals due to the regulation of the between-language activation that bilinguals perform on a daily basis (Bialystok and Majumder, 1998; Bialystok et al., 2004). This advantage in cognitive control has been observed when bilinguals resolve conflict in both linguistic tasks (e.g., Bialystok et al., 2008) and non-linguistic tasks (e.g., Morales et al., 2015). However, the question of whether the bilingual advantage is more a myth than a reality has recently been raised (e.g., Duñabeitia et al., 2013), and it has even been proposed that scientific research was biased and overestimated the benefits associated to bilingualism (e.g., de Bruin et al., 2015). Moreover, data do not seem to support the bilingual advantage in different conflict tasks (e.g., Gathercole et al., 2014).

In this study, we propose that some of the evidence against the “bilingual advantage” stems from the two factors: (a) the possible lack of sensitivity of the measures used to evaluate conflict resolution (i.e., behavioral measures sometimes do not capture differences in conflict resolution between bilinguals and monolinguals, Kousaie and Phillips, 2012), (b) the possible lack of correspondence between the conflict situations that bilinguals resolve on a daily basis (conflict due to the coactivation of linguistic information across languages) and the tasks used to evaluate conflict resolution in bilinguals and monolinguals (conflict tasks that do not involve language processing) (e.g., Flanker tasks).

In our study, we directly addressed these two factors: (a) by introducing both behavioral and electrophysiological measures of conflict resolution in bilinguals and monolinguals, (b) by designing a new linguistic task that would simulate conflict situations that bilinguals experience due to the coactivation between languages.

The rationale behind our study was as follows. If the bilingual advantage in conflict resolution derives from their linguistic experience, that advantage should be observed when resolving conflict in the same cognitive domain; that is, language processing.

In our experiment, we applied the processing of ambiguous words, widely used to investigate the coactivation of languages (i.e., interlingual homographs; Macizo et al., 2010; Martín et al., 2010; Hoshino and Thierry, 2012; Durlik et al., 2016) to the case of ambiguous words within the same language (e.g., within-language homophones) in order to index the possible inhibitory mechanism used to resolve conflict in monolinguals and bilinguals. The behavioral results of our study revealed that all participants experienced conflict due to the coactivation of the two meanings of homophone words. Thus, the participants’ performance was poorer (slower response latency and higher error rate) in the related condition in which the preceding word “agua” (“water” in English) was not related to the subsequent presentation of the orthographic form of the homophone “hola” (“hello” in English) but was related to the alternative orthographic form of the homophone (“ola,” “wave” in English); as compared to the unrelated condition in which neither of the two orthographic forms of the homophone word were related to the preceding word.

This interference effect suggests that the participants activated the two meanings of the ambiguous word, which led to an interference effect when they performed the linguistic task. Therefore, according to studies on language control in monolinguals (Gernsbacher et al., 1990) and bilinguals (Green, 1998), participants would apply inhibition to resolve lexical competition by suppressing the contextually incorrect meaning of the ambiguous word.

At this point, we could question why the practice of bilinguals in the resolution of between-language conflict (e.g., the lexical coactivation of two words, “casa” in Spanish, “house” in English, that lead to the same meaning and may compete for selection in Spanish-English bilinguals) has consequences for the resolution of the within-language conflict evaluated in our study. In our opinion, inhibition is a general cognitive control mechanism that allows the suppression of irrelevant information to perform a given task (for a review of this perspective see Marsh and Anderson, 2022). Thus, the continued practice of bilinguals in between-language conflict resolution would facilitate the processing of within-language conflict evaluated in this work. This approach is supported by studies that (a) show that conflict in one task facilitates the resolution of other types of conflict (Freitas et al., 2007; Kan et al., 2013), and (b) studies that reveal shared underlying neural substrate in tasks that involve the resolution of different types of conflict situations (Peterson et al., 2002, for an fMRI study in which similar neural pattern is observed in the Simon and Stroop task; see also Wu et al., 2020).

Importantly, the behavioral results revealed that the magnitude of the relatedness effect (unrelated minus related condition) was smaller in the bilingual group (37 ms response time, 16% error rate) than in the monolingual group (77 ms response time, 21% error rate). Thus, the latency and accuracy results suggested that bilingual individuals were more efficient than monolinguals in resolving the conflict arising from the coactivation of the homophone irrelevant meaning. As noted in the Section “1. Introduction,” it could be argued that these between-group differences were due to an overall advantage of bilinguals when performing the linguistic task (e.g., an enhanced conflict monitoring skill that would be applied when processing conflict trials and non-conflict trials, Costa et al., 2009). This explanation cannot be ruled out in our study since bilinguals compared to monolinguals displayed faster responses in non-conflict situations (unrelated trials). In addition, between-group differences were larger when participants resolved conflict situations (related trials). Hence, bilinguals processed conflict trials (related trials) more quickly and with fewer errors than monolinguals (165 ms difference and 4.52% difference, respectively); while the magnitude of these between-group differences was smaller in the non-conflict condition (unrelated trials) (125 ms difference and 0.40% difference, respectively). Thus, behavioral outcomes revealed greater efficiency of bilinguals vs. monolinguals in conflict resolution. Taken together, the behavioral pattern might suggest that bilinguals compared to monolinguals displayed superior inhibitory control in language processing and, in addition, greater monitoring of the conflict situations when performing the task (proactive control). In fact, authors such as Hasher et al. (2007) argue that proactive control and inhibition are two critical features of attentional regulation and cognitive control. Specifically, the author proposes that proactive control helps to prepare and maintain a proper attentional setup for the task, whereas the inhibitory mechanism would help to suppress irrelevant information in conflict situations. Thus, previous studies suggest that bilingual experience confers benefits in proactive control (monitoring, Singh and Mishra, 2013) and in the suppressing irrelevant contents in conflict situations (Carlson and Meltzoff, 2008).

On the other hand, it could be claimed that the between-group differences in the processing of ambiguous words were due to a lower quality of lexical representations in bilinguals than in monolinguals (i.e., the lexical quality hypothesis discussed in the Section “1. Introduction,” Hart and Perfetti, 2008). This hypothesis might account for the differences found between bilinguals and monolinguals when processing homographs in L2 (i.e., Paap and Liu, 2014). However, this explanation would be unlikely in our study since all participants performed the task in Spanish, their native language (the L1 of the bilinguals), and bilinguals and monolinguals were matched on Spanish linguistic skills (see Table 1). Consequently, the reduced conflict effect observed in bilinguals versus monolinguals with behavioral measures seems to be due to a more efficient inhibitory control used to resolve conflict. In particular, the irrelevant meaning of the homophone (“ola,” “a wave” in English) would receive more activation in the related condition as it appeared with an associated word (“agua,” “water” in English) compared to the unrelated condition in which the homophone appeared with an unassociated word (e.g., “peluche,” “teddy” in English). Thus, the competition process would be greater in the conflict condition (related trials) than in the non-conflict condition (unrelated trials). If we assume that inhibition is proportional to the degree of lexical competition (e.g., Green, 1998), the type of trial effect would be due to the additional time required to inhibit the irrelevant meaning of the homophone in the related vs. unrelated condition. According to this view, the between-group differences in conflict trials would reflect a more efficient use of this inhibitory mechanism in bilinguals compared to monolinguals.

Concerning electrophysiological data, the results revealed a relatedness effect in all participants, mainly in the N400 time window. However, there were differences between the bilingual and monolingual groups. On the one hand, the differences associated to the relatedness effect (larger positivity in the related condition than in the unrelated condition) appeared earlier in monolinguals (200–300 time window) than in bilinguals (300–400 ms time-window). On the other hand, the relatedness effect was more widely distributed in monolinguals than in bilinguals (see Figure 3). This pattern of results seems to indicate between-groups differences in the degree of semantic activation when participants performed the homophone task. Specifically, the data suggest that the coactivation of semantic information within a language (the two meanings of the homophone) was more efficient in monolinguals than when bilinguals processed them in their L1. This pronounced semantic activation was reflected in the relatedness effect (larger brain wave positivity in the related condition compared to the unrelated condition), which appeared early and was more widely distributed in monolinguals than in bilinguals. In fact, many previous studies about language processing show large brain wave positivity in the N400 time window associated with the ease to access and retrieve semantic contents (see Kutas and Federmeier, 2011, for a review). According to the explanation given in the previous paragraph, this increased semantic activation would lead to greater competition in related vs. unrelated trials.

Importantly, the electrophysiological results also revealed differences between bilinguals and monolinguals associated to conflict resolution. In particular, only the group of monolinguals, in the 400–500 ms time window, showed a greater negativity in the related condition than in the unrelated condition over posterior regions. However, the increased N400 negativity associated to conflict processing appeared to be an overall effect because there were no between-group differences when they were compared in the related and unrelated conditions separately. This pattern of electrophysiological outcomes contrasts with that found in previous studies. For example, Heidlmayr et al. (2015) reported a conflict effect (i.e., Stroop effect) on the N400 amplitude in the 400–500 ms time window. This N400 effect found by these authors was observed in monolinguals but not in bilinguals which could be interpreted in favor of better conflict resolution in bilinguals compared to monolinguals. However, in the Heidlmayr et al. study, it is not clear why bilinguals did not exhibit this electrophysiological effect given that the presence of a larger N400 negativity in conflict vs. non-conflict trials is a relatively well-established finding in the literature for both monolinguals (West and Alain, 1999) and bilinguals (Naylor et al., 2012). Furthermore, in other studies like the one reported by Coderre and van Heuven (2014) no differences were found between monolingual and bilingual in the N400 amplitude associated to conflict resolution in a Stroop task. Specifically, the authors used a Stroop task in which the stimulus onset asynchrony (SOA) of word and color (−400 ms and the standard 0 ms) was manipulated. Behavioral results revealed interference (incongruent vs. neutral condition) in the 0 ms SOA condition, which was of lower magnitude in bilinguals than in monolinguals when performing the task in L2; however, there were no electrophysiological differences between participants in the amplitude of the N400. The greater sensitivity of behavioral versus electrophysiological measures for indexing conflict situations and the effect of bilingual experience may be somewhat problematic, in that electrophysiological measures have greater temporal resolution of the cognitive processes underlying task performance. However, authors such as Heidlmayr et al. suggest the possibility that the absence of N400 Stroop effect might reflect reduced interference that might be due to more efficient inhibition of interfering information (p. 12). Moreover, Hilchey and Klein (2011) conclude that the bilingual advantage in conflict processing is a sporadic and elusive phenomenon (p. 644). Thus, while the behavioral data obtained in our study seem to demonstrate better conflict resolution in bilingual vs. monolingual individuals, the electrophysiological data are not conclusive about the superior conflict resolution related to bilingualism.

Finally, it is important to note that the results obtained in this study should be considered within the framework of the bilingual language experience (see Dussias et al., 2019; De Cat et al., 2023, for reviews). Specifically, not all bilinguals are the same, there would be variability in the amount of cognitive conflict and the underlying cognitive control depending on a multitude of factors such as the amount of exposure to each language, the degree of immersion of bilinguals in each language, the presence or absence of language code switching in the bilinguals’ daily lives, etc. Thus, the generalization of our results to “all bilinguals” should be taken with caution.

## 5. Conclusion

There is an open debate in the scientific literature about the impact that the use of several languages would have on conflict resolution. This possible advantage may depend on the type of bilingual experience and the type of conflict. In our study, behavioral data suggest that bilinguals compared to monolinguals showed better inhibitory control to resolve conflict when they processed lexical ambiguities in their native language. In contrast, electrophysiological data do not provide conclusive confirmation of the possible benefit in language control associated to bilingualism.

## Data availability statement

The datasets presented in this study can be found in online repositories. The names of the repository/repositories and accession number(s) can be found below: https://osf.io/amwn2/.

## Ethics statement

The study was undertaken in accordance with the 1964 Helsinki Declaration and followed the ethical standards delineated by this journal and by the Ethical Committee of the University of Granada (number issued by the Ethical Committee: 957/CEIH/2019). The participants provided their written informed consent to participate in this study.

## Author contributions

FA: formal analysis and writing–reviewing and editing. MR: investigation, formal analysis, visualization, and writing–original draft. PM: conceptualization, methodology, funding acquisition, writing–reviewing and editing, and supervision. All authors contributed to the article and approved the submitted version.
