# Supplementary material for: The impact of bilingualism in within-language conflict resolution: an ERP study
Source: Front Psychol. 2023 May 25;14:1173486. doi: 10.3389/fpsyg.2023.1173486 (PMC10248526; doi:10.3389/fpsyg.2023.1173486)
Supplement: Supplementary file 1 [file Table_1.pdf]

Supplementary Material 1

Homophone Stimuli Used in the Study

Homophone words		Non-homohone words	
Orthographic word 1	Orthographic word 2	Related to Orthographic word 2	Unrelated words
cayado (crook)	callado (silent)	ruidoso (noisy)	película (film)
harte (get tired of)	arte (art)	pintar (paint)	tirantes (braces)
arrollo (mow)	arroyo (stream)	agua (water)	estrella (star)
bienes (assets)	vienes (come)	llegar (arrive)	perro (dog)
baso (base on)	vaso (glasss)	taza (cup)	moda (fashion)
bovina (bovine)	bobina (reel)	caña (cane)	mosaico (mosaic)
hierva (boil)	hierba (grass)	cortar (cut)	borrar (remove)
hunos (huns)	unos (some)	muchos (many)	suelo (floor)
hasta (until)	asta (shaft)	palo (stick)	tono (tone)
hayas (beech)	hallas (find)	perder (lose)	oscuro (dark)
barón (baron)	varón (male)	mujer (woman)	tienda (store)
hola (hello)	ola (wave)	agua (water)	techo (ceiling)
cabe (fit)	cave (dig)	pala (shovel)	blusa (blouse)
habría (there would be)	abría (opened)	cerrar (close)	cascada (waterfall)
vello (fuzz)	bello (beautiful)	feo (ugly)	formato (format)
bello (beautiful)	vello (fuzz)	largo (long)	esfuerzo (effort)
ola (wave)	hola (hello)	saludo (greeting)	peluche (cuddly toy)
desecho (rubbish)	deshecho (damaged)	arreglar (fix)	textura (texture)
horcas (gallows)	orcas (killer whales)	pescado (fish)	siempre (always)
vienes (come)	bienes (assets)	valor (value)	espalda (back)
cayo (cay)	callo (corn)	comida (food)	oscuro (dark)
hala (wow)	ala (wing)	volar (fly)	mágico (magic)
vota (vote)	bota (boot)	patada (kick)	lado (side)
vallas (fences)	vayas (go)	parar (stop)	móvil (mobile)
onda (wave)	honda (sling)	tiro (shot)	foto (photo)
asta (shaft)	hasta (until)	tarde (late)	traición (betrayal)
hato (herd)	ato (tie)	nudo (knot)	bebé (baby)

hecho (done)	echo (throw)	pelota (ball)	gracias (thank)
cave (dig)	cabe (fit)	ajustado (tight)	saltar (jump)
hierba (grass)	hierva (boil)	caliente (hot)	diente (tooth)
arroyo (stream)	arrollo (mow)	coger (take)	grapa (staple)
vayas (go)	bayas (berries)	fruta (fruit)	grifo (tap)
has (have / has)	as (ace)	poker (poker)	mono (monkey)
sabia (wise woman)	savia (sap)	pegajoso (sticky)	casa (house)
vote (vote)	bote (small boat)	embarcar (load)	ventana (window)
callado (silent)	cayado (crook)	madera (wood)	papel (paper)
basto (coarse)	vasto (vast)	amplio (large)	tarjeta (card)
maya (mayan)	malla (mesh)	voleibol (volleyball)	cielo (heaven)
tuvo (had)	tubo (pipe)	manguera (hose)	pinza (clothes peg)
echo (throw)	hecho (done)	completado (finished)	ratón (mouse)

Note. Experimental material used in the study. Each experimental trial was composed by a homophone word and a non-homophone word. Each homophone had two orthographic forms (Orthographic word 1 and Orthographic word 2 columns). In the related condition, the orthographic word 1 was paired with a word semantically related to the alternative orthographic form of the homophone (related to orthographic word 2). In the unrelated condition, the orthographic word 1 appeared along with an unrelated word.
