# Supplementary material for: The impact of bilingualism in within-language conflict resolution: an ERP study
Source: Front Psychol. 2023 May 25;14:1173486. doi: 10.3389/fpsyg.2023.1173486 (PMC10248526; doi:10.3389/fpsyg.2023.1173486)
Supplement: Supplementary file 2 [file Table_2.pdf]

Supplementary Material 2

Statistical Analyses Performed on ERP Data. Relatedness Effects and Interactions in the Homophone Task Performed by Bilinguals

Time-window	Effects	*F*	*p*
200-300 ms	Relatedness	0.02	.88
	Relatedness x AP axis	0.72	.47
	Relatedness x LM axis	0.51	.55
	Relatedness x AP axis x LM axis	0.30	.85
300-400 ms	Relatedness	3.55	.07
	Relatedness x AP axis	0.38	.65
	Relatedness x LM axis	1.36	.26
	Relatedness x AP axis x LM axis	0.19	.93
400-500 ms	Relatedness	3.75	.06
	Relatedness x AP axis	0.20	.73
	Relatedness x LM axis	1.84	.17
	Relatedness x AP axis x LM axis	1.45	.21

Note. AP: Anterior-Posterior, LM: Lateral-Medial. *$p \leq .05$, **$p \leq .001$
