# Supplementary material for: The impact of bilingualism in within-language conflict resolution: an ERP study
Source: Front Psychol. 2023 May 25;14:1173486. doi: 10.3389/fpsyg.2023.1173486 (PMC10248526; doi:10.3389/fpsyg.2023.1173486)
Supplement: Supplementary file 3 [file Table_3.pdf]

Supplementary Material 3

Statistical Analyses Performed on ERP Data. Relatedness Effects and Interactions in the Homophone Task Performed by Monolinguals

Time-window	Effects	*F*	*p*
200-300 ms	Relatedness	0.88	.36
	Relatedness x AP axis	2.11	.15
	Relatedness x LM axis	1.61	.21
	Relatedness x AP axis x LM axis	1.70	.10
300-400 ms	Relatedness	6.42	.02*
	Relatedness x AP axis	4.54	.03*
	Relatedness x LM axis	3.06	.06
	Relatedness x AP axis x LM axis	2.83	.02*
400-500 ms	Relatedness	2.53	.12
	Relatedness x AP axis	6.46	.01*
	Relatedness x LM axis	2.99	.08
	Relatedness x AP axis x LM axis	2.60	.05*

Note. AP: Anterior-Posterior, LM: Lateral-Medial. $*p \leq .05$, $**p \leq .001$
