# Supplementary material for: The impact of bilingualism in within-language conflict resolution: an ERP study
Source: Front Psychol. 2023 May 25;14:1173486. doi: 10.3389/fpsyg.2023.1173486 (PMC10248526; doi:10.3389/fpsyg.2023.1173486)

Supplementary Material 4

Grand Average ERP Obtained in the Related and Unrelated Condition in the Bilingual Group

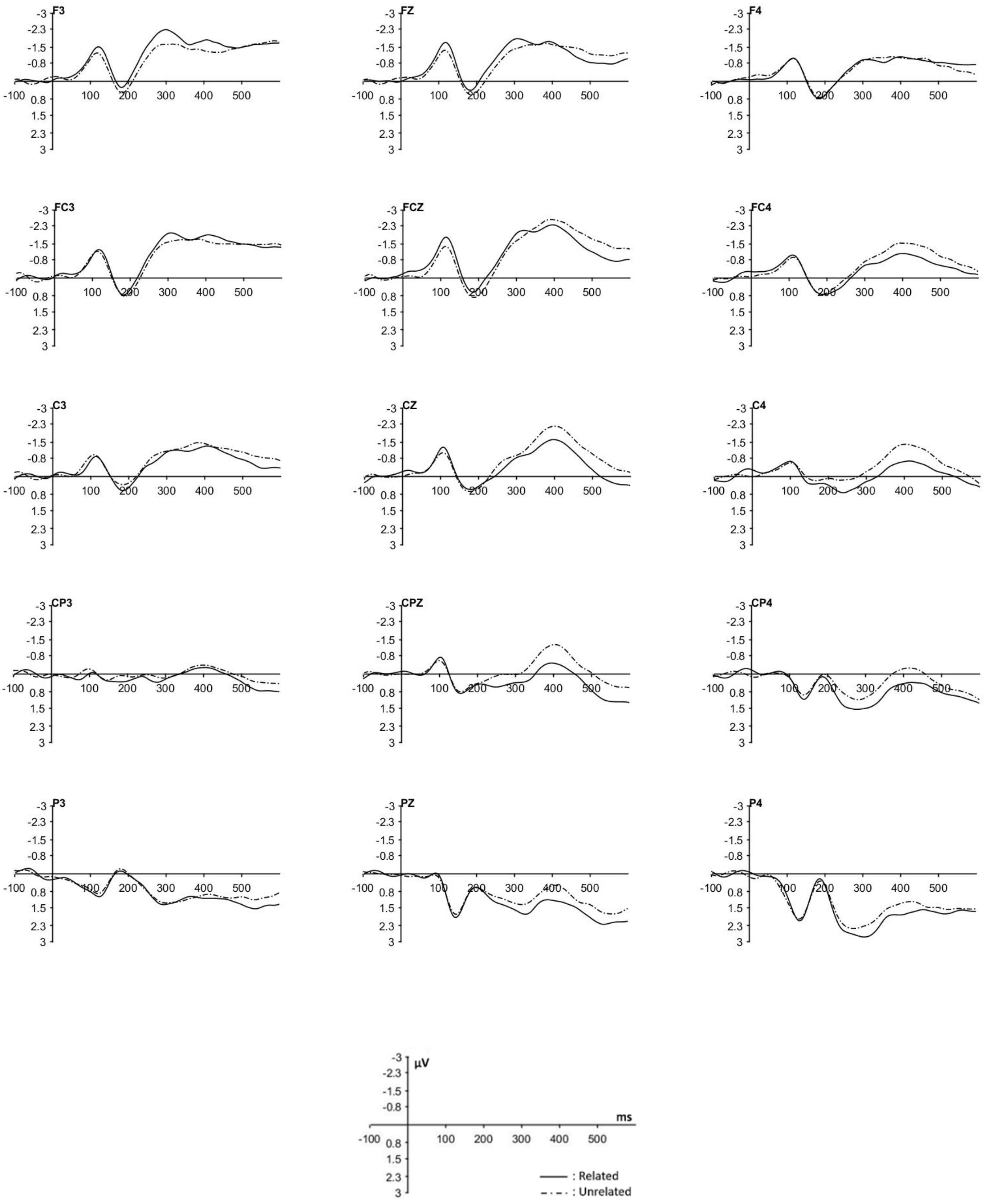

Supplement: Supplementary file 4 [file Image_1.pdf]
