# Supplementary material for: The impact of bilingualism in within-language conflict resolution: an ERP study
Source: Front Psychol. 2023 May 25;14:1173486. doi: 10.3389/fpsyg.2023.1173486 (PMC10248526; doi:10.3389/fpsyg.2023.1173486)

Supplementary Material 5

Grand Average ERP Obtained in the Related and Unrelated Condition in the Monolingual Group

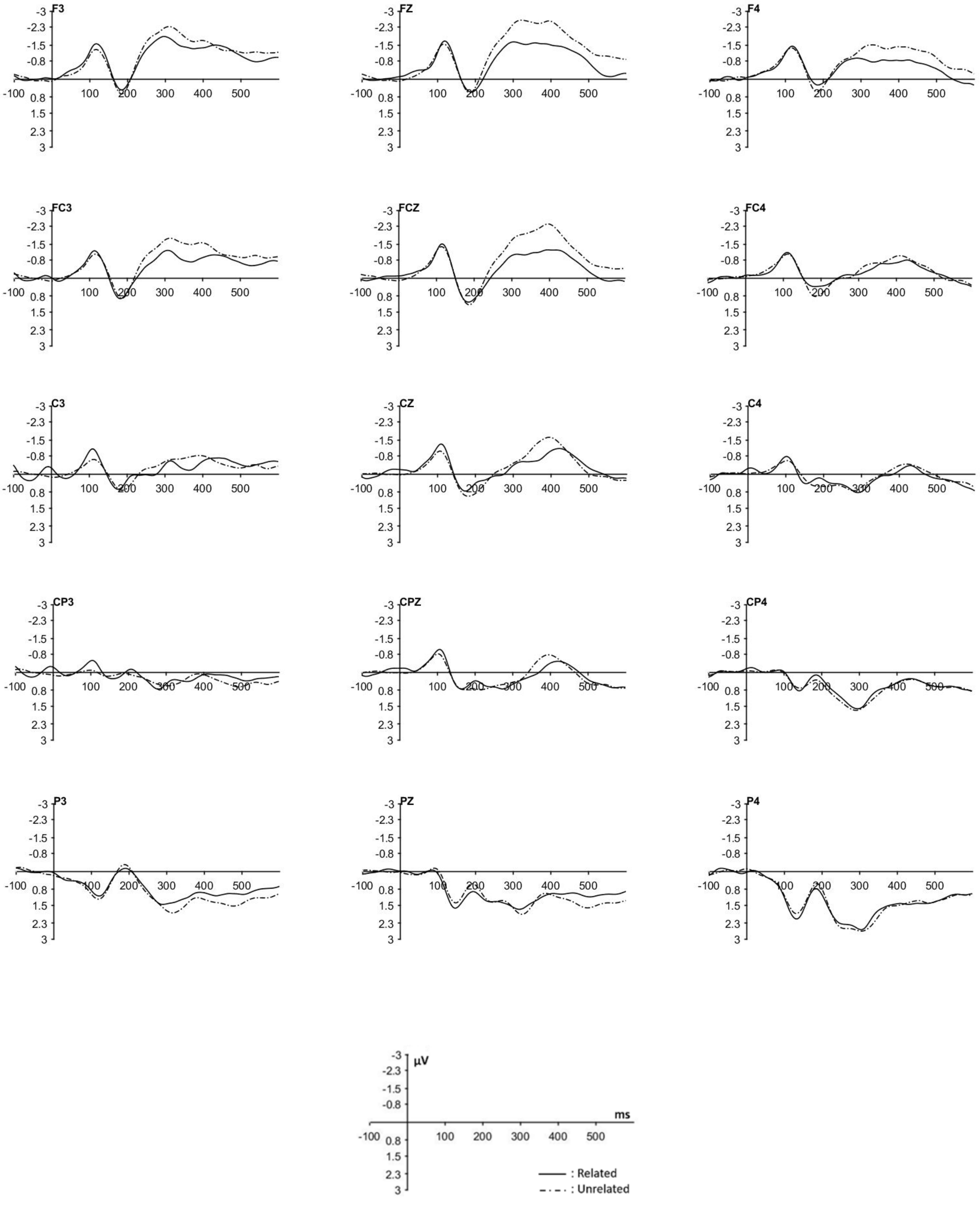

Supplement: Supplementary file 5 [file Image_2.pdf]
