# Supplementary material for: The impact of bilingualism in within-language conflict resolution: an ERP study
Source: Front Psychol. 2023 May 25;14:1173486. doi: 10.3389/fpsyg.2023.1173486 (PMC10248526; doi:10.3389/fpsyg.2023.1173486)
Supplement: Supplementary file 6 [file Table_4.pdf]

Supplementary Material 6: The Relationship Between LEAP-Q Measures and Group Membership

To explore which measures of the LEAP-Q questionnaire were related to the group membership, we ran logistic regressions with group as the dependent variable (which was coded as follows: 0 for bilinguals and 1 for monolinguals) and LEAP-Q measures as independent variables (which were scaled to compare the estimates between different measures). In the table below, we present the coefficients associated to each of the LEAP-Q measures. If the estimates are negative, the higher the probability of belonging to the bilingual group. Almost all the estimates for the measures in L2 were significant and in an expected direction. Only three items, *interacting with friends in L2*, *interacting with family in L2*, and *listening to the radio in L2* were not significant. This makes sense, as all the bilinguals were native Spanish speakers currently living in Spain and mainly interacting with Spanish peers. On the other hand, *listening to the radio* might be an obsolete item. Moreover, the *Speech fluency and Proficiency in L2* had the largest estimates and were the most relevant predictors of the group membership.

Unsurprisingly, some predictors from the LEAP-Q regarding L1 were also significant. This was the case for *Speech fluency and Proficiency in L1* (whose estimates were also negative). Bilinguals rated themselves higher on these L1 measures, and this could be due to myriad of factors. However, note that these estimates were lower than any other significant predictor in L2. Additionally, four more items were significant, these were *Exposure to L1, Preference for reading, Speaking,* and *Watching TV in L1* (whose estimates were positive). This makes sense as these measures are inversely proportional to the measures in L2. Simply put, bilinguals must divide their time for activities such as reading, speaking, or watching TV in their first and second language.

We see that these comparisons are largely similar to the *t*-test comparisons in Table 1 of the manuscript, but we have also quantified which LEAP-Q measures were the most relevant for our sample, and which could best divide our sample into bilinguals and monolingual beside the language certificates that they obtained. These would be the items *Speech fluency in L2* and *Proficiency in L2*.

Table 1. *Coefficients from the logistic regression analyses with the LEAP-Q predictors regarding L2 and group membership (bilingual, monolingual) as a dependent variable.*

**Predictor**	**Estimate**	**Std. error**	***z* value**	***p* value**
L2 Speech Fluency	-3.17	0.76	-4.15	< .000
L2 Speech Comprehension	-2.09	0.54	-3.86	< .000
L2 Reading Proficiency	-2.35	0.63	-3.7	< .000
L2 Proficiency	-3.58	0.94	-3.82	< .000
L2 Exposure	-1.21	0.46	-2.62	.009
L2 Reading Preference	-1.88	0.56	-3.37	.001
L2 Speaking Preference	-0.99	0.39	-2.57	.010
L2 Interacting with Friends	-0.47	0.29	-1.65	.100
L2 Interacting with Family	-0.16	0.28	-0.56	.575
L2 Reading	-0.51	0.28	-1.81	.070
L2 TV	-1.19	0.34	-3.52	< .000
L2 Radio	-0.26	0.27	-0.98	.325

Table 2. *Coefficients from the logistic regression analyses with the LEAP-Q predictors regarding L1 and group membership (bilingual, monolingual) as a dependent variable.*

**Predictor**	**Estimate**	**Std. error**	***z* value**	***p* value**
L1 Speech Fluency	-0.9	0.32	-2.84	.004
L1 Speech Comprehension	-0.36	0.27	-1.34	.179
L1 Reading Proficiency	-0.21	0.26	-0.8	.424
L1 Proficiency	-0.57	0.28	-2.02	.043
L1 Exposure	0.71	0.34	2.05	.040
L1 Reading Preference	1.37	0.42	3.27	.001
L1 Speaking Preference	0.91	0.34	2.67	.008
L1 Interacting with Friends	0.74	0.42	1.74	.082
L1 Interacting with Family	0.37	0.33	1.12	.262
L1 Reading	0.56	0.3	1.87	.061
L1 TV	0.68	0.32	2.16	.031
L1 Radio	0.38	0.27	1.41	.160
