# Supplementary material for: The impact of bilingualism in within-language conflict resolution: an ERP study
Source: Front Psychol. 2023 May 25;14:1173486. doi: 10.3389/fpsyg.2023.1173486 (PMC10248526; doi:10.3389/fpsyg.2023.1173486)
Supplement: Supplementary file 7 [file Table_5.pdf]

Supplementary material 7: L2 measures as continuous variables in the bilingual group.

	RT				RT				RT			
Predictors	*Estimates*	*SE*	*Statistic*	*p*	*Estimates*	*SE*	*Statistic*	*p*	*Estimates*	*SE*	*Statistic*	*p*
(Intercept)	959.56 ***	33.01	29.07	**<0.001**	959.43 ***	32.85	29.20	**<0.001**	959.22 ***	32.89	29.16	**<0.001**
L2 Speech Fluency	9.70	32.66	0.30	0.766								
Condition	41.93 ***	10.17	4.12	**<0.001**	41.93 ***	10.20	4.11	**<0.001**	41.94 ***	10.20	4.11	**<0.001**
L2 Speech Fluency x Condition	-5.74	10.15	-0.57	0.572								
L2 Speech Comprehension					20.08	32.56	0.62	0.538				
L2 Speech Comprehension x Condition					-2.23	10.16	-0.22	0.826				
L2 Reading Proficiency									-18.29	32.94	-0.56	0.579
L2 Reading Proficiency x Condition									-1.27	10.18	-0.12	0.901
**Random Effects**												
σ^2	42698.91				42701.66				42702.07			
τ_{00}	914.15 $_{stimuli}$				912.52 $_{stimuli}$				912.92 $_{stimuli}$			
	33500.62 $_{subject.1}$				33181.57 $_{subject.1}$				33257.81 $_{subject.1}$			
τ_{11}	787.09 $_{subject.cond.cod}$				805.90 $_{subject.cond.cod}$				806.47 $_{subject.cond.cod}$			
ρ_{01}												
ρ_{01}												
ICC	0.03				0.03				0.03			
N	32 $_{subject}$				32 $_{subject}$				32 $_{subject}$			
	40 $_{stimuli}$				40 $_{stimuli}$				40 $_{stimuli}$			
Observations	2217				2217				2217			
Marginal R^2 / Conditional R^2	0.012 / 0.037				0.019 / 0.044				0.017 / 0.042			

* $p<0.05$ ** $p<0.01$ *** $p<0.001$

	RT				RT				RT			
Predictors	*Estimates*	*SE*	*Statistic*	*p*	*Estimates*	*SE*	*Statistic*	*p*	*Estimates*	*SE*	*Statistic*	*p*
(Intercept)	959.49 ***	33.03	29.05	**<0.001**	962.23 ***	33.69	28.56	**<0.001**	962.24 ***	33.33	28.87	**<0.001**
L2 Proficiency	6.90	32.97	0.21	0.834								
Condition	41.93 ***	10.19	4.11	**<0.001**	44.20 ***	9.77	4.52	**<0.001**	44.23 ***	10.24	4.32	**<0.001**
L2 Proficiency x Condition	-3.65	10.16	-0.36	0.720								
L2 Exposure					22.36	33.13	0.67	0.500				
L2 Exposure x Condition					16.41	9.77	1.68	0.093				
L2 Reading Preference									-35.66	33.07	-1.08	0.281
L2 Reading Preferencen x Condition									2.89	10.22	0.28	0.778
**Random Effects**												
σ^2	42700.45				42908.70				42902.08			
τ_{00}	913.61 stimuli				892.72 stimuli				890.67 stimuli			
	33541.56 subject.1				33848.92 subject.1				33115.86 subject.1			
τ_{11}	800.95 subject.cond.cod				419.15 subject.cond.cod				706.49 subject.cond.cod			
ρ_{01}												
ρ_{01}												
ICC	0.03				0.02				0.02			
N	32 subject				31 subject				31 subject			
	40 stimuli				40 stimuli				40 stimuli			
Observations	2217				2143				2143			
Marginal R^2 / Conditional R^2	0.011 / 0.036				0.022 / 0.044				0.039 / 0.062			

* *p<0.05* ** *p<0.01* *** *p<0.001*

	RT				RT				RT			
Predictors	*Estimates*	*SE*	*Statistic*	*p*	*Estimates*	*SE*	*Statistic*	*p*	*Estimates*	*SE*	*Statistic*	*p*
(Intercept)	949.07 ***	31.85	29.79	**<0.001**	958.77 ***	31.81	30.14	**<0.001**	959.53 ***	33.01	29.07	**<0.001**
L2 Speaking Preference	-30.16	31.53	-0.96	0.339								
Condition	40.76 ***	9.73	4.19	**<0.001**	41.92 ***	10.06	4.17	**<0.001**	41.92 ***	10.19	4.11	**<0.001**
L2 Speaking Preference x Condition	13.27	9.70	1.37	0.171								
L2 Interacting with Friends					49.66	31.09	1.60	0.110				
L2 Interacting with Friends x Condition					-10.21	10.01	-1.02	0.308				
L2 Interacting with Family									-9.11	32.31	-0.28	0.778
L2 Interacting with Family x Condition									2.74	10.13	0.27	0.787
**Random Effects**												
σ^2	39977.16				42702.57				42702.75			
τ_{00}	715.89 $_{stimuli}$				906.17 $_{stimuli}$				911.86 $_{stimuli}$			
	29316.51 $_{subject.1}$				31016.89 $_{subject.1}$				33495.05 $_{subject.1}$			
τ_{11}	487.37 $_{subject.cond.cod}$				716.36 $_{subject.cond.cod}$				799.60 $_{subject.cond.cod}$			
ρ_{01}												
ρ_{01}												
ICC	0.02				0.02				0.03			
N	30 $_{subject}$				32 $_{subject}$				32 $_{subject}$			
	40 $_{stimuli}$				40 $_{stimuli}$				40 $_{stimuli}$			
Observations	2083				2217				2217			
Marginal R^2 / Conditional R^2	0.033 / 0.053				0.064 / 0.087				0.012 / 0.037			

* *p<0.05* ** *p<0.01* *** *p<0.001*

	RT				RT				RT			
Predictors	*Estimates*	*SE*	*Statistic*	*p*	*Estimates*	*SE*	*Statistic*	*p*	*Estimates*	*SE*	*Statistic*	*p*
(Intercept)	959.45 ***	32.47	29.54	**<0.001**	959.44 ***	32.97	29.10	**<0.001**	959.27 ***	32.79	29.26	**<0.001**
L2 Reading	-34.52	32.10	-1.08	0.282								
Condition	41.93 ***	10.20	4.11	**<0.001**	41.93 ***	10.20	4.11	**<0.001**	41.93 ***	10.17	4.12	**<0.001**
L2 Reading x Condition	-1.89	10.18	-0.19	0.852								
L2 TV					12.71	32.76	0.39	0.698				
L2 TV x Condition					-0.28	10.17	-0.03	0.978				
L2 Radio									23.37	32.73	0.71	0.475
L2 Radio x Condition									-5.05	10.15	-0.50	0.619
**Random Effects**												
σ^2	42702.82				42702.58				42701.98			
τ_{00}	911.45 $_{stimuli}$				912.03 $_{stimuli}$				911.92 $_{stimuli}$			
	32387.51 $_{subject.1}$				33422.86 $_{subject.1}$				33042.34 $_{subject.1}$			
τ_{11}	804.78 $_{subject.cond.cod}$				807.43 $_{subject.cond.cod}$				784.93 $_{subject.cond.cod}$			
ρ_{01}												
ρ_{01}												
ICC	0.03				0.03				0.03			
N	32 $_{subject}$				32 $_{subject}$				32 $_{subject}$			
	40 $_{stimuli}$				40 $_{stimuli}$				40 $_{stimuli}$			
Observations	2217				2217				2217			
Marginal R^2 / Conditional R^2	0.035 / 0.060				0.013 / 0.039				0.022 / 0.047			

* *p<0.05* ** *p<0.01* *** *p<0.001*
