# Supplementary material for: The impact of bilingualism in within-language conflict resolution: an ERP study
Source: Front Psychol. 2023 May 25;14:1173486. doi: 10.3389/fpsyg.2023.1173486 (PMC10248526; doi:10.3389/fpsyg.2023.1173486)
Supplement: Supplementary file 8 [file Table_6.pdf]

Supplementary material 8: L2 measures as continuous variables in the monolingual group.

	RT				RT				RT			
Predictors	*Estimates*	*SE*	*Statistic*	*p*	*Estimates*	*SE*	*Statistic*	*p*	*Estimates*	*SE*	*Statistic*	*p*
(Intercept)	1107.32 ***	35.76	30.96	**<0.001**	1107.58 ***	36.17	30.62	**<0.001**	1107.44 ***	36.29	30.52	**<0.001**
L2 Speech Fluency	-32.62	35.28	-0.92	0.355								
Condition	82.79 ***	12.13	6.82	**<0.001**	82.82 ***	12.13	6.83	**<0.001**	82.84 ***	12.13	6.83	**<0.001**
L2 Speech Fluency x Condition	1.43	12.09	0.12	0.906								
L2 Speech Comprehension					-16.19	36.02	-0.45	0.653				
L2 Speech Comprehension x Condition					6.02	12.05	0.50	0.617				
L2 Reading Proficiency									4.20	35.98	0.12	0.907
L2 Reading Proficiency x Condition									4.50	12.06	0.37	0.709
**Random Effects**												
σ^2	67565.03				67554.49				67559.29			
τ_{00}	1573.76 $_{stimuli}$				1576.83 $_{stimuli}$				1575.60 $_{stimuli}$			
	33675.41 $_{subject}$				34491.73 $_{subject}$				34737.08 $_{subject}$			
ICC	0.34				0.35				0.35			
N	28 $_{subject}$				28 $_{subject}$				28 $_{subject}$			
	40 $_{stimuli}$				40 $_{stimuli}$				40 $_{stimuli}$			
Observations	1899				1899				1899			
Marginal R^2 / Conditional R^2	0.026 / 0.360				0.019 / 0.360				0.016 / 0.360			

* *p<0.05* ** *p<0.01* *** *p<0.001*

	RT				RT				RT			
Predictors	*Estimates*	*SE*	*Statistic*	*p*	*Estimates*	*SE*	*Statistic*	*p*	*Estimates*	*SE*	*Statistic*	*p*
(Intercept)	1107.52 ***	36.16	30.63	**<0.001**	1116.02 ***	37.58	29.70	**<0.001**	1108.64 ***	36.13	30.69	**<0.001**
L2 Proficiency	-16.46	35.80	-0.46	0.646								
Condition	82.81 ***	12.13	6.83	**<0.001**	80.09 ***	12.80	6.26	**<0.001**	86.21 ***	12.36	6.97	**<0.001**
L2 Proficiency x Condition	4.70	12.07	0.39	0.697								
L2 Exposure					30.94	36.74	0.84	0.400				
L2 Exposure x Condition					6.25	12.81	0.49	0.626				
L2 Reading Preference									73.17 *	35.49	2.06	**0.039**
L2 Reading Preferencen x Condition									6.89	12.37	0.56	0.578
**Random Effects**												
σ^2	67558.88				69575.01				62418.75			
τ_{00}	1575.73 $_{stimuli}$				1380.37 $_{stimuli}$				1319.78 $_{stimuli}$			
	34470.78 $_{subject}$				34750.19 $_{subject}$				30835.06 $_{subject}$			
ICC	0.35				0.34				0.34			
N	28 $_{subject}$				26 $_{subject}$				25 $_{subject}$			
	40 $_{stimuli}$				40 $_{stimuli}$				40 $_{stimuli}$			
Observations	1899				1756				1690			
Marginal R^2 / Conditional R^2	0.019 / 0.360				0.023 / 0.357				0.068 / 0.385			

* *p<0.05* ** *p<0.01* *** *p<0.001*

	**RT**				**RT**				**RT**			
Predictors	*Estimates*	*SE*	*Statistic*	*p*	*Estimates*	*SE*	*Statistic*	*p*	*Estimates*	*SE*	*Statistic*	*p*
(Intercept)	1116.11 ***	38.05	29.34	**<0.001**	1107.57 ***	35.71	31.01	**<0.001**	1107.54 ***	33.74	32.83	**<0.001**
L2 Speaking Preference	6.02	37.92	0.16	0.874								
Condition	80.07 ***	12.80	6.26	**<0.001**	82.98 ***	12.12	6.84	**<0.001**	82.71 ***	12.13	6.82	**<0.001**
L2 Speaking Preference x Condition	17.79	12.71	1.40	0.162								
L2 Interacting with Friends					36.46	35.17	1.04	0.300				
L2 Interacting with Friends x Condition					19.49	12.11	1.61	0.108				
L2 Interacting with Family									72.86 *	34.06	2.14	**0.033**
L2 Interacting with Family x Condition									6.24	11.99	0.52	0.603
**Random Effects**												
σ^2	69505.40				67465.27				67557.33			
τ_{00}	1379.71 $_{stimuli}$				1582.82 $_{stimuli}$				1571.29 $_{stimuli}$			
	35673.70 $_{subject}$				33575.34 $_{subject}$				29733.57 $_{subject}$			
ICC	0.35				0.34				0.32			
N	26 $_{subject}$				28 $_{subject}$				28 $_{subject}$			
	40 $_{stimuli}$				40 $_{stimuli}$				40 $_{stimuli}$			
Observations	1756				1899				1899			
Marginal R^2 / Conditional R^2	0.016 / 0.358				0.028 / 0.361				0.066 / 0.362			

* *p<0.05* ** *p<0.01* *** *p<0.001*

	RT				RT				RT			
Predictors	*Estimates*	*SE*	*Statistic*	*p*	*Estimates*	*SE*	*Statistic*	*p*	*Estimates*	*SE*	*Statistic*	*p*
(Intercept)	1107.57 ***	36.30	30.51	**<0.001**	1107.66 ***	36.32	30.50	**<0.001**	1107.32 ***	36.13	30.65	**<0.001**
L2 Reading	-3.70	35.65	-0.10	0.917								
Condition	82.89 ***	12.13	6.83	**<0.001**	82.96 ***	12.13	6.84	**<0.001**	82.88 ***	12.13	6.83	**<0.001**
L2 Reading x Condition	7.50	12.09	0.62	0.535								
L2 TV					3.82	35.97	0.11	0.915				
L2 TV x Condition					17.26	12.04	1.43	0.152				
L2 Radio									19.41	35.70	0.54	0.587
L2 Radio x Condition									9.65	12.05	0.80	0.423
**Random Effects**												
σ^2	67546.94				67482.69				67538.08			
τ_{00}	1581.55 $_{stimuli}$				1587.33 $_{stimuli}$				1581.55 $_{stimuli}$			
	34755.67 $_{subject}$				34791.04 $_{subject}$				34405.20 $_{subject}$			
ICC	0.35				0.35				0.35			
N	28 $_{subject}$				28 $_{subject}$				28 $_{subject}$			
	40 $_{stimuli}$				40 $_{stimuli}$				40 $_{stimuli}$			
Observations	1899				1899				1899			
Marginal R^2 / Conditional R^2	0.016 / 0.360				0.017 / 0.361				0.019 / 0.360			

* *p*<0.05 ** *p*<0.01 *** *p*<0.001
